# Circular RNA CircNOLC1, Upregulated by NF-KappaB, Promotes the Progression of Prostate Cancer via miR-647/PAQR4 Axis

**DOI:** 10.3389/fcell.2020.624764

**Published:** 2021-01-08

**Authors:** Wenbin Chen, Shengren Cen, Xumin Zhou, Taowei Yang, Kaihui Wu, Libin Zou, Junqi Luo, Chuanyin Li, Daojun Lv, Xiangming Mao

**Affiliations:** ^1^Department of Urology, Zhujiang Hospital, Southern Medical University, Guangzhou, China; ^2^Guangdong Key Laboratory of Urology, Department of Urology, The First Affiliated Hospital of Guangzhou Medical University, Guangzhou, China

**Keywords:** circRNAs, prostate cancer, miR-647, PAQR4, NF-kappaB, progression

## Abstract

**Background:**

CircRNAs recently have shown critical roles in tumor biology. However, their roles in prostate cancer (PCa) remains largely unclear.

**Methods:**

CircRNA microarrays were performed in immortal prostate cell line RWPE1 and PCa cell lines as DU145, PC3, LNCaP, C4-2, and 22RV1. Combined with upregulated circRNAs in PCa tissues, circNOLC1 expression was validated in PCa cells and tissues *via* qRT-PCR and FISH. Sanger sequencing, actinomycin D, gDNA, and cDNA, RNase R assays were used to assess the circular characteristics of circNOLC1. CCK-8, colony formation, transwell migration assays, and mice xenograft models were conducted to evaluate the functions of PCa cells after circNOLC1 knockdown and overexpression. RNA pulldown, luciferase reporter assay, FISH (fluorescence *in situ* hybridization), and CHIP were utilized to illustrate the further mechanisms of circNOLC1.

**Results:**

Our research indicated that circNOLC1 was overexpressed in PCa cells and tissues, and circNOLC1 was more stable than linear NOLC1 mRNA. CircNOLC1 promoted PCa cells proliferation and migration *in vitro* and *vivo*. Additionally, we found that circNOLC1 could upregulate PAQR4 expression by sponging miR-647, leading to the activation of PI3K/Akt pathway. Moreover, NF-kappaB was identified to bind to the NOLC1 promoter sites and upregulated both NOLC1 and circNOLC1 expression.

**Conclusion:**

CircNOLC1, elevated by transcription factor NF-kappaB, promotes PCa progression *via* a miR-647/PAQR4 axis, and circNOLC1 is a potential biomarker and target for PCa treatment.

## Introduction

Prostate cancer (PCa) is currently the first and second leading cause of new diagnosis and mortality, accounting for 21% of all new cancer cases and 10% of all death cases in men (Siegel et al., 2020). Although primary tumors frequently can be cured through androgen deprivation therapy (Berger et al., 2011), many patients exhibited poor prognosis due to tumor metastasis (Wang et al., 2018). Therefore, further researches are needed to explore the novel diagnostic and therapeutic biomarkers and reveal the detailed underlying mechanisms in PCa progression.

Circular RNA (CircRNA), a novel type of non-coding RNA, is formed by covalently closed loop structures without free terminal ends (Chen, 2020). Due to their special property, circRNAs show higher stability and stronger resistance to ribonuclease R degradation than linear RNAs (Chen and Yang, 2015). Owing to the progression of advanced high-throughput sequencing, various circRNAs were discovered to exhibit abnormal expression in cancers (Chen et al., 2019b; Vo et al., 2019). Recent reports revealed that circRNAs exert their biological functions mainly relying on the miRNA sponging (Hansen et al., 2011, 2013). Besides, circRNAs could also play a role in interacting with RNA binding proteins (RBPs) (Liu et al., 2020). In PCa, circRNAs, such as, circ_0007494 and circFMN2, have been recognized to function as competing endogenous RNAs (ceRNAs) to relieve the inhibition of miRNA targets (Shan et al., 2020; Zhang et al., 2020). CircAR3 was identified to show high expression levels in the plasma of PCa patients, which may serve as a novel PCa biomarker (Luo et al., 2019). These findings revealed the significant roles of circRNAs in PCa. However, there are still plenty of circRNAs involved in the PCa have not been identified.

In this study, circRNA microarray in five common PCa cells combined with the published microarray data for PCa tissues, we identified 22 different circRNAs. Subsequently, circNOLC1 (circBase ID: has_circ_0000257) were chosen for further study to explore its role in the PCa proliferation and progression by using a series of *in vitro* assays and *in vivo* mouse model. Finally, the underlying mechanism of circNOLC1 were also evaluated. In summary, our works revealed that circNOLC1 could act as a oncogene in the progression of PCa, and may provide a novel target for the diagnosis and treatment of PCa.

## Materials and Methods

### Clinical Specimens

Eighty prostate tissues and sixteen adjacent tissues were obtained from patients who underwent radical prostatectomy (RP) at Zhujiang Hospital of Southern Medical University from 2016 to 2018, and then the paraffin specimens were constructed as a tissue microarray (TMA). The clinical details of patients were collected from the medical records. All experimental procedures were approved by the Ethics Committee of the Southern Medical University Zhujiang Hospital. The median age of the enrolled patients was 67.5 years and average age was 65.1 (range: 20–97 years). Clinical TNM staging and Gleason scores of patient specimens were based on the American Joint Committee on Cancer (AJCC) Eighth Edition (2017) and the 2016 World Health Organization (WHO) classification of genitourinary tumors. The detailed clinicopathological information of all samples was presented in Supplementary Table 1.

### CircRNA Microarrays

Arraystar Human circRNA Array v2 (Kangcheng Biotech, Shanghai, China) was applied to analysis circRNA microarray. Total RNA from each sample was quantified with the NanoDrop ND-1000. Sample preparation and microarray hybridization were performed as outlined in the standard protocols stipulated by Arraystar, as described previously (Chen et al., 2019a). Normalized Intensity of each group (averaged normalized intensities of replicate samples, log2 transformed) were analyzed by paired *t*-test (*P*: 0.05). Quantile normalization and subsequent data processing was performed through the R 4.0.2 software limma package. Differentially expressed circRNAs were identified via Fold Change filtering. Hierarchical Clustering was used to perform the distinguishable circRNAs expression pattern among the samples.

### Bioinformatics Analysis

The circRNA-miRNA interactome was drawn by circBank^1^ and circinteractome^2^. Overlapping candidates were considered as putative miRNA targets. Two algorithms (TargetScan and miRpathDB) were used to predict the potential miRNAs targeting the 3’-UTR of PAQR4. Data from The Cancer Genome Atlas (TCGA) was analyzed by Starbase 2.0^3^.

### Cell Culture and Transfection

DU145, PC3, C4-2, LNCaP, 22RV1 (PCa cell lines), RWPE1 (normal prostate epithelial cell line), and HEK-293T cells were obtained from Cell Bank of Chinese Academy of Sciences, grown with RPMI-1640 medium (Gibco, United States) supplemented with 10% FBS (Gibco, United States) and incubated at 37°C in 5% CO_2_. Small interfering RNAs (siRNAs) of circNOLC1 and NF-kappaB, and miR-647 inhibitor or mimics were purchased from RiboBio Company (Guangzhou, China). Lentivirus vectors (GeneChem Bio-Medical Biotechnology, Shanghai, China) were utilized to establish cell lines stably overexpressing circNOLC1, and the transfected cells were selected in puromycin (2 μg/ml) for 1 week. All the target sequences were shown in Supplementary Table 2.

### Cellular Fraction and RNA Isolation

Nuclear and Cytoplasmic Extraction Reagents (No. 78833, Thermo Fisher Scientific, United States) were used to separate nuclear and cytoplasm of cultured cells following the manufacturer’s protocol. And RNA extraction was described as followed.

### RNA Extraction and RT-qPCR

Total RNA was obtained from cells with TRIzol (Invitrogen, United States), while the cDNA was reverse-transcribed via utilizing PrimeScript RT reagent Kit (TaKaRa) following the manufacturer’s instructions. The genome DNA was isolated by special extraction kit (DP304, TIANGEN, China). SYBR Green PCR Master Mix (TaKaRa) and Applied Bio-systems 7500 Fast Real-Time RCR System (Applied Biosystems, United States) were used for RT-qPCR analysis. Data were acquired from three independent experiments and normalized to GAPDH. All the primers were shown in Supplementary Table 2.

### Actinomycin D and RNase R Treatment Assay

To compare the stability of linear RNA and circRNA, Actinomycin D (MedChemExpress, China) was added into medium to block RNA transcription and the solvent dimethyl sulfoxide (DMSO; Sigma) was applied as a negative control. Cells were treated with actinomycin D or DMSO in a final concentration of 1 μg/mL for 0, 4, 8, 12, and 24 h, and then the RNA was extracted for RT-qPCR detection, using 18S as an internal reference. For RNase R treatment, 2 mg total RNA was incubated for 15 min at 37°C with or without 3 U/mg RNase R (No. R0301, Geneseed, China), and followed by RT-qPCR analysis.

### Fluorescence *in situ* Hybridization (FISH) Analysis

CircNOLC1 and miR-647 were captured by Cy3-labeled probes (RiboBio, Guangzhou, China) and Alexa 488-labeled probes (FOCOFISH, Guangzhou, China). FISH experiment was conducted using Fluorescent *in situ* Hybridization Kit (No. C10910, RiboBio, Guangzhou, China), according to the official guidelines. DAPI was utilized to stain the nuclei. Subsequently, circNOLC1 and miR-647 were observed through a confocal microscope (LSM 880 with Airyscan, Carl Zeiss, Germany).

### Cell Proliferation Measurement

Cell proliferation was measured by CCK-8 kit (CK-04, Dojindo). 2,000 PCa cells were planted into 96-well plates. Then medium with 10%CCK-8 was added into each well, and incubated for 2 h. Subsequently, the absorbance values at 450 nm were detected by a microplate reader (EXL800, BioTek Instruments).

### Colony Formation Assay

PCa cells were seeded into 6-well plates and cultured with complete medium for 2 weeks. The formed cells were fixed with 4% paraformaldehyde for 10 min and stained with Giemsa (Baso Diagnostics Inc, Zhu Hai, China) for 5 min. Each experiment was performed with three replicates.

### Transwell Migration Assays

Cell migration assay was performed using transwell inserts (8 mm pores; Corning, NY, United States) in 24-well plates. PCa cells (5 × 10^4^) were resuspended in 300 μl serum-free medium and seeded into the upper chamber of the insert. Subsequently, the lower chamber was filled with 500 μl complete medium. After 24 h, the cells were fixed with 4% paraformaldehyde for 10 min and stained with Giemsa (Baso Diagnostics Inc, Zhu Hai, China). Five randomly selected fields were photographed using an invert microscope at 200× magnification. The cell numbers of each image were counted by Image J software. Experiments were performed in triplicate.

### Western Blot

Cells were lysed in RIPA lysis buffer (#KGP250, KeyGEN BioTECH, Nanjing, China), following the manufacturer’s instructions. Equal amounts of proteins were separated in 10% SDS-PAGE gels and transferred to PVDF membranes (Millipore, Germany). The membranes were then blocked for 1 h with 5% no fat milk and incubated overnight at 4°C with the following primary antibodies: EMT marker (#9782, Cell signaling, United States), anti-PAQR4 (#13401-1-AP, Proteintech), anti-β-actin (#60008-1-Ig, Proteintech). Subsequently, the membranes were immersed in the anti-rabbit or anti-mouse secondary antibodies (#7074S or #7076S, Cell signaling, United States) for 1.5 h at room temperature. Enhanced chemiluminescence (ECL) kit (Pierce Biotechnology, Rockford, IL, United States) was used to detect and visualize the protein.

### RNA-Pulldown

CircRNA pulldown assay was carried out using Pierce^TM^ Magnetic RNA-Protein Pull-Down Kit (No: 20164, Thermo Fisher Scientific, United States), all procedures were followed as manufacturer’s instructions. Then the final RNA was extracted by TRIzol (Invitrogen, United States) and analyzed by RT-qPCR.

### Dual-Luciferase Reporter Assay

The sequences of circNOLC1 and its mutant types without miR-647 binding sites were designed and packaged into pEZX-MT06 vector (GeneCopoeia, Guangzhou, China), termed as circNOLC1-WT and circNOLC1-MUT. The miRNA mimics were purchased from RiboBio Company (Guangzhou, China). HEK-293T cells (5 × 10^5^) were seeded into each well of a 12-well plate for 24 h. Then, a mixture of luciferase reporter vectors and miRNA mimics was transfected into cells. The relative luciferase activity was measured utilizing Luc-Pair^TM^ Duo-Luciferase HS Assay Kit (GeneCopoeia, China). Each group was confirmed in triplicate.

### Xenograft Model

All experimental animal procedures were authorized by the Animal Care and Use Committee of Southern Medical University and Specific Pathogen Free (SPF) conditions were used to raise animals. BALB/c nude mice were obtained from the Animal Center of Southern Medical University, Guangzhou, China. A total of 2 × 10^6^ du145 cells stably transfected with circNOCL1 or Vector were subcutaneously injected into the mice axillae (*n* = 6 per group). Tumor volume was measured every 4–5 days and volumes were calculated using the formula: length × width^2^ × 0.5.

### Immunohistochemistry Analysis

Detection of Ki-67 and CD31 was performed on 3 μm thick paraffin sections with the indicated antibodies. Briefly, sections were incubated with primary antibodies Ki-67 (1:250, ab15580, Abcam, United States) or CD31 (ZM-0044, Zhong Shan Jin Qiao, Beijing, China) antibodies at 4°C overnight. Subsequently, Horseradish peroxidase (HRP)-conjugated secondary antibodies (dilution 1:250, Beyotime, Shanghai, China) were further incubated at 37°C for 30 min. Finally, the sections were stained with 3,3’-diaminobenzidine (DAB, ZLI-9018, Zhong Shan Jin Qiao, Beijing, China) for 5 min and examined with a microscope.

### CHIP Assay

CHIP assay was performed using SimpleChIP^®^ Plus Enzymatic Chromatin IP Kit (#9005, Cell Signaling Technology, United States), according to the manufacturer’s protocols. Anti-NF-kappaB antibody for CHIP was obtained from Cell Signaling Technology (#8242). The detailed binding sites between the promoter sites of NOLC1 and NF-kappaB were predicted by *Consite*^4^, as: GGGAAGTCCC. The specific primers for binding sites were shown in Supplementary Table 2. And the following analysis was detected by RT-qPCR. Experiments were performed in triplicate.

### Statistical Analysis

All statistical analysis was performed using GraphPad Prism 8 (GraphPad Software, United States). The data were presented as mean ± *SD*. Two-tailed Student’s *t*-test or ANOVA was conducted to analyze the significance between variables. The relation between circNOLC1 expression and clinicopathological properties was analyzed using a χ^2^ test. *P* < 0.05 was considered as statistically significant.

## Results

### Upregulation and Characterization of CircNOLC1 in PCa

**FIGURE 1:**
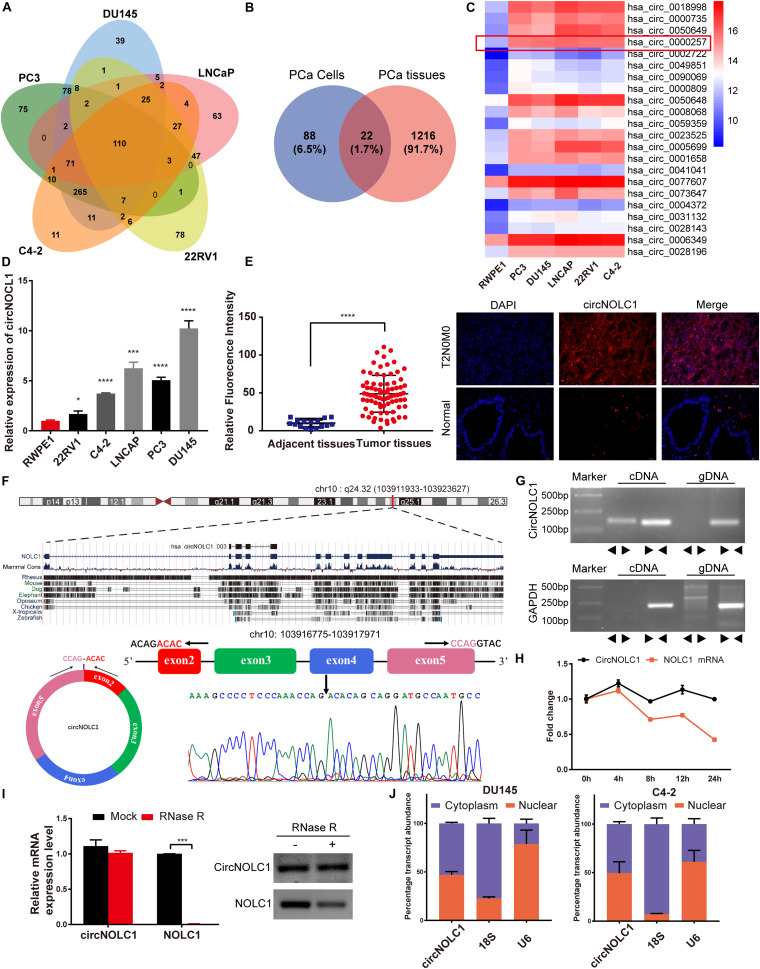
Screening and characterization of circNOLC1 in PCa. **(A,B)** Schematic illustration demonstrates that 22 circRNAs were commonly upregulated in PCa cells and tissues. **(C)** The heatmap showing upregulated circRNA expression profiles between PCa cells and normal prostate cells. **(D)** Expression of circNOLC1 in five PCa cells and normal prostate cells. **(E)** Relative Fluorescence Intensity of each tissues and representative images of FISH analysis of circNOLC1 in normal and PCa tissues. Scale bar = 50 μm. **(F)** Sketch map for circNOLC1 and sanger sequencing confirming the back splicing site of circNOLC1. **(G)** The existence of circNOLC1 was validated by qRT-PCR and Gel electrophoresis. Divergent primers could amplify circNOLC1 in cDNA but not gDNA. GAPDH was used as negative control. **(H,I)** Actinomycin D treatment and RNase R to confirm the circular characteristics of circNOLC1. **(J)** Cellular localization of circNOLC1 in DU145 and C4-2 cells. Nuclear and cytoplasmic fraction was separated followed by RNA extraction. circNOLC1, U6 and 18S levels were analyzed by qRT-PCR. *p < 0.05, ***p < 0.001, ****p < 0.0001.

To investigate the differential expressed circRNAs in PCa, we performed a microarray of the PCa cell lines, DU145, PC3, LNCaP, C4-2, and 22RV1 cells (Supplementary Figure 1A). We found 22 circRNAs both upregulated in five PCa cells and a microarray dataset that contains five pairs of high-grade and low-grade PCa (Figures 1A–C). The expression levels of top five upregulated circRNAs in PCa cells and normal prostate cell were validated via qRT-PCR (Figure 1D and Supplementary Figures 1B–E), circNOLC1 (hsa_circ_0000257) displayed the greatest increase and was chosen for further study. Next, we verified the expression of circNOLC1 in 79 Prostate adenocarcinoma tissues and 16 normal prostate tissues by FISH assays (Figure 1E and Table 1). These findings demonstrated that circNOLC1 is upregulated in PCa cells and tissues.

**TABLE 1:** Expression of circNOLC1 in normal prostate tissues and prostate cancer tissues (*n* = 79).

	Group	*N*	CircNOLC1		χ^2^	*p*-values
			Low	High		
Type	Normal	16	16	0	46.039	< 0.001
	Adenocarcinoma	79	12	67		
Age	≤ 69	41	6	35	0.036	0.849
	> 69	38	5	33		
Clinical stage	I–II	35	2	33	1.059	0.303
	III–IV	22	3	19		
Primary tumor	T1–T2	35	2	33	1.059	0.303
	T3–T4	22	3	19		
Gleason score	≤ 6	13	2	11	0.0004	> 0.9999
	≥ 7	66	10	56		

**CircNOLC1 expression was determined by FISH; p-value is from χ^2^-test. A remarkably increasing frequency of positive expression of circNOLC1 was detected in prostate cancer specimens compared to normal prostate tissues (P < 0.001, χ^2^-test).**

CircNOLC1 is generated by head-to-tail splicing of NOLC1 exon 2–5 and contains 487 nucleotides (Figure 1F, left panel, upper). Sanger sequencing was used to confirm the putative circNOLC1 junction (Figure 1F, right panel, lower). Besides, we amplified circNOLC1 and NOLC1 from cDNA and gDNA utilizing divergent and convergent primers, which showed that circNOLC1 only exist in the cDNA (Figure 1G). Actinomycin D assay demonstrated that circNOLC1 was more stable compared with linear NOLC1 (Figure 1H). Moreover, RNase R assays exhibited that the linear mRNA NOLC1 was digested, while circNOLC1 not (Figure 1I). We also isolated the cytoplasmic and nuclear RNA in DU145 and C4-2 cells, which demonstrated that circNOLC1 was expressed equally in cytoplasm and nuclear (Figure 1J). These results illustrated the circular characteristics of circNOLC1.

### CircNOLC1 Promotes PCa Proliferation and Migration *in vitro*

**FIGURE 2:**
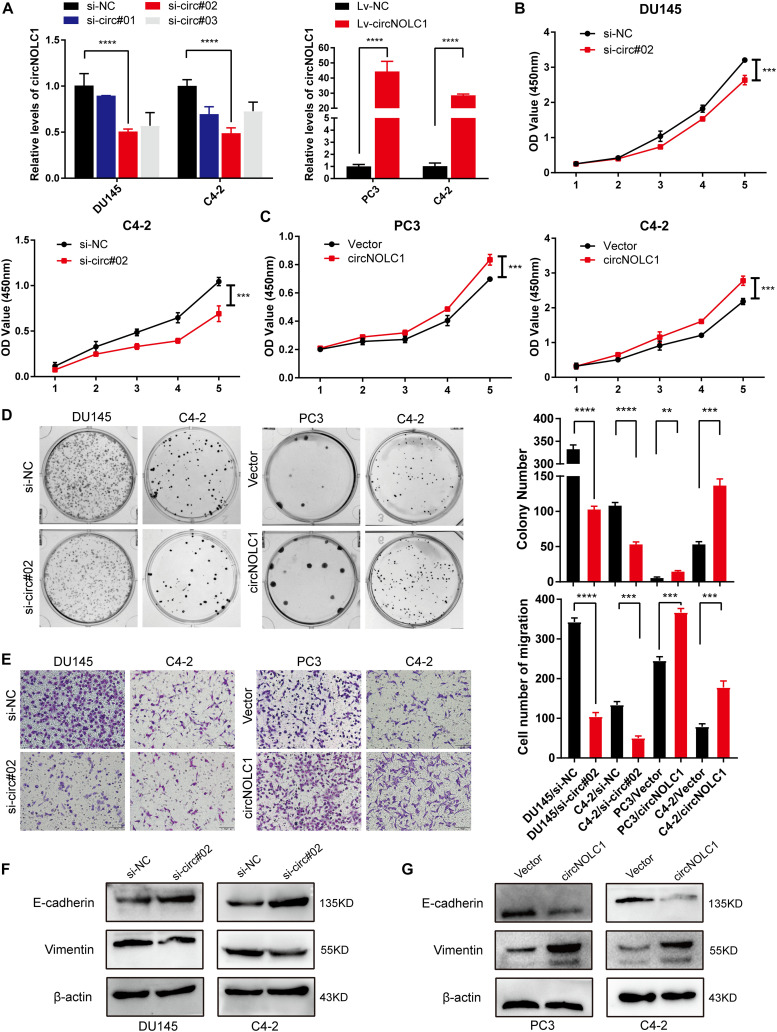
CircNOLC1 promotes cell growth and migration in PCa cells. **(A)** RT-qPCR was performed to confirm the inhibiting efficiency of three siRNAs targeting circNOLC1 in DU145 and C4-2 cells and the overexpression of circNOLC1 vector in PC3 and C4-2 cells. **(B–D)** CCK8 and colony formation assays were utilized to evaluate the proliferation of PCa cells after knockdown and overexpression of circNOLC1. **(E)** Transwell migration assay was conducted to assess the cell migration ability in circNOLC1-silenced and circNOLC1-overexpressed PCa cells. **(F,G)** The influence of circNOLC1 on the protein expression of Vimentin and E-cadherin were measured by western blot. **p < 0.01, ***p < 0.001, ****p < 0.0001.

To discover the function of circNOL1 in PCa, we randomly chose DU145 and C4-2 cell lines for silencing, PC3 and C4-2 for overexpression. We constructed three siRNAs targeting circNOLC1 to silence its expression. Finally, si-circ#02 exhibited the highest silencing efficiency measured by qRT-PCR and was chosen for the following experiments (Figure 2A, left). Meanwhile, we established stably overexpressed circNOLC1 in PC3 and C4-2 cells via utilizing lentiviral vectors, and the circNOLC1 overexpression cells exhibited a great increased level of circNOLC1 (Figure 2A, right). However, the NOLC1 mRNA level was not affected by circNOLC1 expression (Supplementary Figures 2A,B). CCK-8 and colony formation assays exhibited that the cell proliferation ability was restrained after circNOLC1 knockdown, while the overexpression of circNOLC1 showed the opposite results (Figures 2B–D). Transwell migration assays demonstrated that circNOLC1 downregulation significantly decreased PCa cells migration, while circNOLC1 overexpression revealed the opposite effects (Figure 2E). Meanwhile, western blots exhibited that loss of circNOLC1 significantly decreased E-cadherin expression and increased Vimentin level, while gain of circNOLC1 showed the opposite results (Figure 2F). Besides, CCK-8 and Transwell migration assays revealed that knockdown of NOLC1 also suppressed the growth and migration of PCa cells (Supplementary Figures 2C–E). These results revealed that circNOLC1 promotes PCa progression *in vitro*.

### CircNOLC1 Acted as a miR-647 Sponge in PCa Cells

**FIGURE 3:**
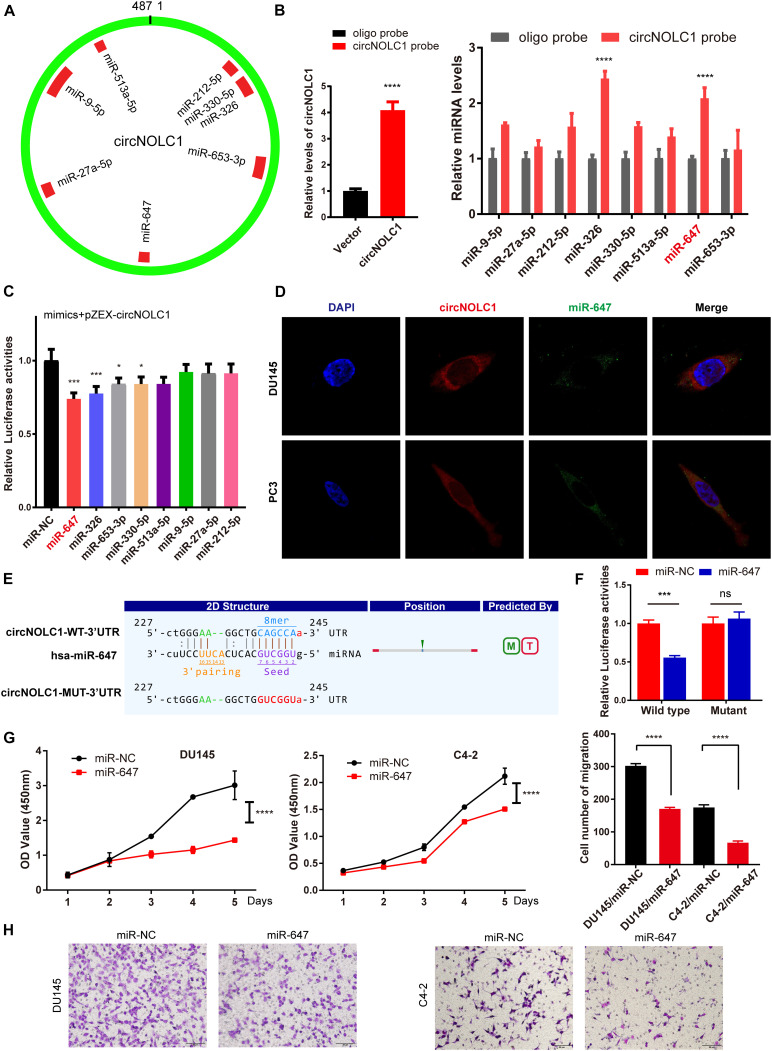
CircNOLC1 acts as a sponge of miR-647. **(A)** Schematic illustration exhibiting the potential circNOLC1 targeting miRNAs, predicted by miRanda, Targetscan, and CircInteractome. **(B)** RNA-Pulldown was performed in DU145 cells using circNOLC1 and negative control probes. **(C)** Effects of nine predicted miRNAs and a negative control on the luciferase activity of circNOLC1, as determined by luciferase reporter assay. **(D)** Colocalization between circNOLC1 and miR-647 was observed using RNA-FISH in DU145 and PC3 cells. The nuclei were stained with DAPI. Scale bar = 40 μm. **(E)** Schematic illustration exhibiting the potential binding sites between circNOLC1 and miR-647, predicted by Targetscan. **(F)** The luciferase activity of pEZX-circNOLC1 in HEK-293 T cells subsequently co-transfected with miR-647. **(G,H)** CCK8 assays and transwell assays were utilized to detect the proliferation and migration of PCa cells after overexpression of miR-647. *p < 0.05, ***p < 0.001, ****p < 0.0001.

Plenty of reports have revealed that circRNAs mainly function as miRNA sponges in various cancers (Hansen et al., 2013; Zheng et al., 2016). To figure out how circNOLC1 play a role in PCa, we predicted the potential miRNA targets of circNOLC1 using three databases (including miRanda, TargetScan, and CircInteractome). We chose eight miRNAs overlapped in the three databases for further study (Figure 3A). To verify the interaction between circNOLC1 and miRNAs, circRNA pulldown assay was performed in DU145 cells via designed circNOLC1 probe. The observation showed that circNOLC1, miR-647 and miR-326 were significantly enriched (Figure 3B). Besides, Luciferase-circNOLC1 reporters were transfected into HEK-293T cells along with miRNA mimics or negative controls, which indicated that miR-647 significantly reduced luciferase activity, compared to miR-NC (Figure 3C). Next, RNA-FISH was utilized to confirm the localization of circNOLC1 and miR-647 in DU145 and PC3 cells. Notably, circNOLC1 and miR-647 were mainly co-localized in the cytoplasm of PCa cells (Figure 3D). Moreover, luciferase reporter assays were constructed in DU145 cells by transfected with luciferase reporter vectors (including the wild type or mutant sequence targeted by miR-647). The luciferase activity was obviously decreased in cells co-transfected with the miR-647 mimics and the wild-type sequence, when compared with the mutant sequence (Figures 3E,F). Taken together, we provided evidences that circNOLC1 could directly bind to miR-647 in PCa cells.

### CircNOLC1 Upregulates PAQR4 Expression Through Sponging miR-647

**FIGURE 4:**
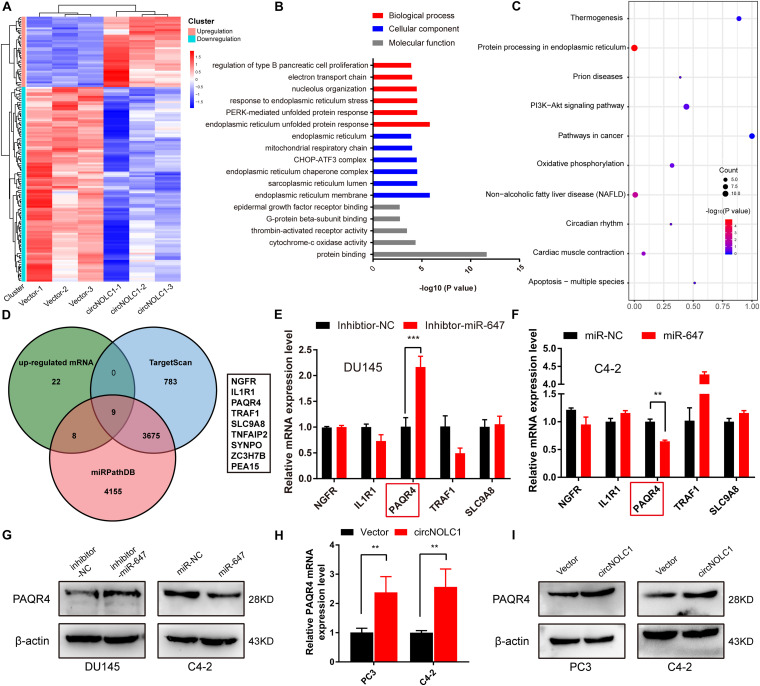
CircNOLC1 upregulates the PAQR4 level through sponging miR-647. **(A)** The heatmap of differentially expressed mRNAs in PC3 cells transfected with Vector or circNOLC1. Each sample was mixed with three replicates. **(B,C)** GO and KEGG analyses of DEGs in PC3 cells. **(D)** Venn diagram illustrating overlapping of miR-647 targeting mRNAs and upregulated mRNAs in PC3 cells. **(E,F)** The mRNA expressions of NGFR, IL1R1, PAQR4, TRAF1, and SLC9A8 were measured via RT-qPCR. **(G)** Relative PAQR4 protein levels in PCa cells transfected with miR-647 inhibitor or mimics. **(H,I)** PAQR4 mRNA and protein expression in circNOLC1 overexpression PCa cells. **p < 0.01; ***p < 0.001.

Due to previous reports, miR-647 could retard tumor progression via inhibiting downstream oncogenes (Ye et al., 2017; Qin et al., 2020). We subsequently discovered that overexpression of miR-647 promoted PCa cells proliferation and migration, which indicated that miR-647 act as a tumor inhibitor role in PCa (Figures 3G,H). Therefore, we assumed that circNOCL1 drove tumor development by avoiding downregulation of these oncogenes induced by miR-647. We performed RNA-seq in PC3-circNOLC1 and PC3-Vector cells, and discovered that 39 genes were upregulated and 108 genes were downregulated in PC3-circNOLC1 cells compared with PC3-Vector cells (Figure 4A). GO analysis showed the mainly enriching GO terms (Figure 4B), KEGG analysis were mainly enriched in Pathways in cancer, PI3K-Akt signaling pathway, and apoptosis (Figure 4C). Next, TargetScan and miRPathDB were utilized to predict the downstream targets of miR-647, nine overlapped genes were obtained from the 39 upregulated and miR-647 targets genes (Figure 4D). We picked up the top five upregulated genes for further studies, as NGFG, IL1R1, PAQR4, TRAF1, and SLC9A8. Then, we, respectively, transfected miR-647 inhibitor and mimics into DU145 and C4-2 cells. The five chosen genes mRNA levels were verified by qRT-PCR, and the results exhibited that PAQR4 mRNA level was remarkably increased after miR-647 knockdown and decreased after miR-647 overexpression (Figures 4E,F). Meanwhile, PAQR4 protein level showed the same expression change with mRNA (Figure 4G). Moreover, PAQR4 mRNA and protein levels were significantly upregulated in circNOLC1 overexpression PCa cells (Figure 4H).

### NF-KappaB Is an Upstream TF of NOLC1 in PCa Cells

**FIGURE 5:**
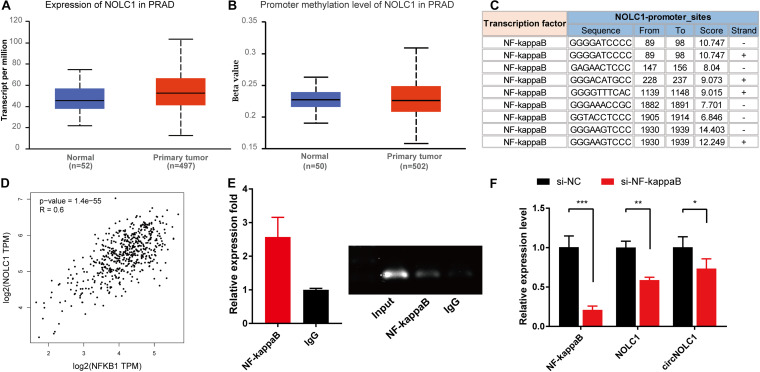
NF-kappaB is an upstream transcription factor of NOLC1 in PCa cells. **(A,B)** The expression and promoter methylation level of NOLC1 in PCa via TCGA database. **(C)** Potential binding sites between NF-kappaB and NOLC1 promoter sites was predicted by CONSITE. **(D)** The relationship between NF-kappaB and NOLC1 expression levels in TCGA, analyzed by GEPIA. **(E)** ChIP-PCR validated the direct binding site of NF-kappaB to the NOLC1 promoter. **(F)** NF-kappaB, NOLC1, and circNOLC1 mRNA levels after NF-kappaB knockdown in DU145 cells, measured by RT-qPCR. *p < 0.05, **p < 0.01, ***p < 0.001.

Previous studies showed that the expression of circRNA was tightly associated with its linear mRNA. To investigate the abnormal expression of circNOLC1 in PCa, we analyzed the bioinformation of NOLC1 in PCa from TCGA database. We found that NOLC1 was upregulated in PCa and the promoter methlation level of NOLC1 showed no difference between the primary tumor and normal group (Figures 5A,B). We then predicted the potential TFs of NOLC1 by *Consite*, and found that NF-kappaB scores ranked the most (Figure 5C). NF-kappaB was obviously correlated with NOLC1 expression (Figure 5D). Moreover, we confirmed the direct binding between NF-kappaB and NOLC1 promoter sites via CHIP assay (Figure 5E). NOLC1 expression levels were notably decreased due to the reduction of NF-kappaB, while circNOLC1 exhibited the same trend (Figure 5F). These results revealed that NF-kappaB could regulate the expression of NOLC1, thus leading to a downregulation of circNOLC1.

### CircNOLC1 Promotes PCa Tumor Growth *in vivo*

**FIGURE 6:**
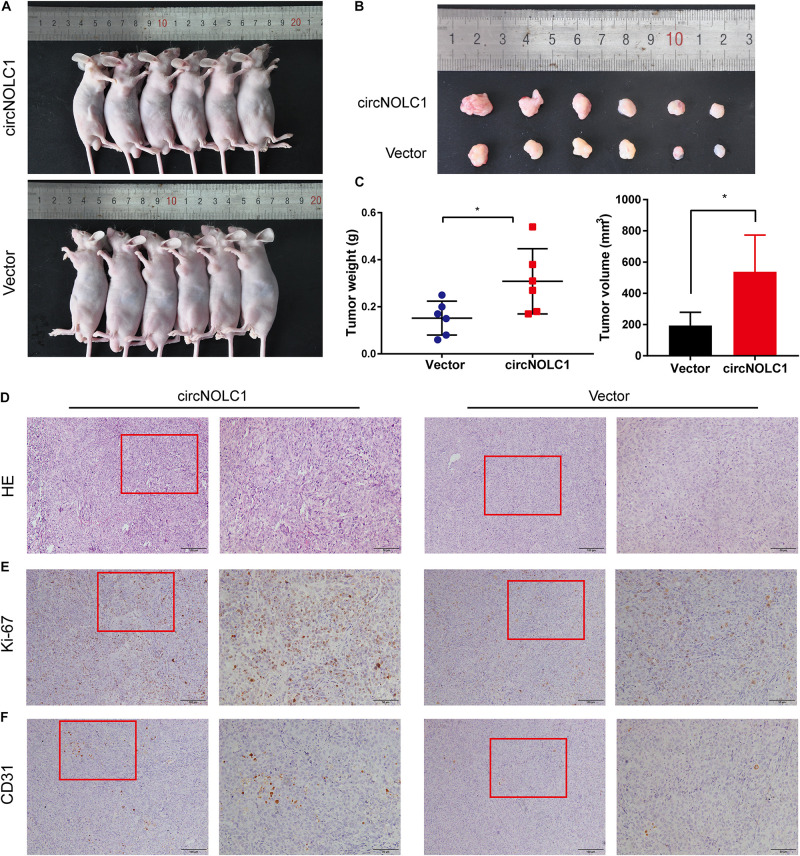
CircNOLC1 promotes the growth of PCa cells *in vivo*. **(A,B)** Images of subcutaneous xenograft tumors derived from DU145 cells transfected with vector and circNOLC1. **(C)** Tumor weights and volume of DU145 cells were shown. **(D)** H&E staining of xenograft tumors. **(E,F)** Ki-67 and CD31 expression in xenograft tumors were detected by IHC. *p < 0.05.

To investigate the functions of circNOLC1 *in vivo*, DU145 cells transfected with vector and circNOLC1 were subcutaneously injected in nude mice. Tumor volumes were measured 5 days followed injection. The mice were euthanized 4 weeks after injection, the subcutaneous tumor tissues were separated, and the weight and volume of isolated tumors were measured. As the results showed, overexpression of circNOLC1 remarkably increased tumors growth *in vivo* (Figures 6A–C). The histopathological features of the isolated tumor tissues were revealed by H&E staining (Figure 6D). Besides, the expression of Ki-67 antigen in circNOLC1-overexpression xenografts tumors was significantly increased, which was detected by IHC (Figure 6E). Moreover, IHC results of the tumor tissues also revealed that CD31 expression was higher in circNOLC1-overexpression tumor compared to the vector, which indicated that circNOLC1 promoted tumor angiogenesis *in vivo* (Figure 6F). These results demonstrated that circNOLC1 overexpression drove PCa tumor growth *in vivo*.

## Discussion

Recent studies have shown non-coding RNAs play a growing role in PCa (Martens-Uzunova et al., 2014; Hua et al., 2019). CircRNAs, a novel non-coding circular RNAs, were once considered as junks of the transcription product. Due to the development of NGS, numerous circRNAs were explored and participated in the biological functions of tumor development, especially in PCa (Chen et al., 2019b; Yang et al., 2019). For instance, circMBOAT2 sponged miR-1271-5p to promote PCa progression (Shi et al., 2020); circSMAD2 restrained miR-9 to govern PCa migration (Han et al., 2019); circFMN2 accelerated PCa tumorigenesis via a miR-1238/LHX2 axis (Shan et al., 2020). Therefore, it is of great clinical value to identify potential early biomarkers for diagnosis and prognosis.

Here, circRNA microarrays were established in five PCa cells as DU145, PC3, LNCaP, C4-2, 22RV1, compared with immortalized normal prostatic epithelium (RWPE1). We discovered 110 upregulated circRNAs both in five PCa cells, and 22 were overlapped overexpressing in PCa tissues. Among them, top five upregulated circRNAs were chosen for further validation, and circNOLC1 was significantly elevated in PCa cells and tissues. Meanwhile, circNOLC1 exhibited stronger stability than its linear mRNA. Through *in vivo* and *in vitro* experiments, circNOLC1 was confirmed to induce cell proliferation and migration, which indicated that it may act as an oncogene in PCa.

Nucleolar and coiled-body phosphoprotein 1 (NOLC1), the host gene of circNOLC1, was firstly discovered as a nuclear localization signal binding protein, which functions as a chaperone that shuttles between the nucleolus and cytoplasm (Meier and Blobel, 1990, 1992). Previous researches have illustrated that NOLC1 is overexpressed in nasopharyngeal carcinomas and regulate tumorigenesis by working with TP53 (Hwang et al., 2009). Besides, NOLC1 exhibited a low expression in hepatocellular carcinoma tissues and overexpression of NOLC1 inhibited hepatocellular carcinoma cells proliferation (Duan et al., 2013; Yuan et al., 2017). Interestingly, a recent study of mass spectrometry-based proteomics have revealed that NOLC1 is significantly increased in PCa samples and cell lines (Kwon et al., 2020). Our studies demonstrated that NOLC1 could promote PCa cells proliferation and migration, however, the detailed mechanisms of its oncogenic roles still needed to be fully investigated.

Previous studies indicated that circRNAs exert their functions by various biological processes, such as miRNA sponges, protein-binding, and transcriptional and translational regulation (Kristensen et al., 2019). The ceRNA mechanism suggested that circRNAs competitively bind to miRNA to relieve the suppression on miRNA targeting genes (Zhong et al., 2018). Deng et al. (2020) indicated that circRHOBTB3 suppress gastric cancer growth by sponging miR-654-3p; Li et al. (2018) revealed that circITGA7 act as a ceRNA of miR-370-3p to inhibit RAS pathway, thus restraining CRC development. Here, our results indicated that circNOLC1 were co-localized mainly in the cytoplasm of PCa cells which implied that it may act as a ceRNA. Thus, we screened and validated the interaction between circNOLC1 and miRNAs by circRNA pulldown and dual-luciferase reporter assay, results demonstrated that circNOLC1 function as a sponge of miR-647. Meanwhile, miR-647 was identified as a tumor inhibitor in glioma and gastric cancer (Ye et al., 2017; Qin et al., 2020). Whereas, its role in PCa remains unclear. We identified that miR-647 act as a tumor inhibitor in PCa. Through the overlap of miR-647 target prediction and RNA-seq in PC-3 cells with overexpression of circNOLC1, we acquired nine potential miR-647 target genes. Then, top five candidate target mRNAs were validated the *via* knockdown and overexpression of miR-647, and PAQR4 was confirmed to be the most likely downstream target gene. Further immunoblot validated that PAQR4 was negatively regulated by miR-647. These findings supported that circNOLC1 sponges with miR-647 promoted PCa progression *via* upregulating PAQR4 expression.

Progestin and adipoQ receptor family member 4 (PAQR4) was reported to be high expression in PCa cells and tissues, and significantly improve PCa malignant phenotype by activating PI3K/Akt pathway (Ye et al., 2020). Besides, Zhang et al. (2018) demonstrated that PAQR4 exert its oncogenic role in breast cancer through inhibiting CDK4 degradation. Moreover, PAQR4 promoted cell growth, metastasis, and chemoresistance in non-small-cell lung cancer (Wu and Liu, 2019; Xu et al., 2020). Combing to our KEGG results of RNA-seq in circNOLC1 overexpression, we assumed that circNOLC1 activates PI3K/Akt pathway in PCa *via* a miR-647/PAQR4 axis.

The mechanism of circRNAs biogenesis is a remarkably complicated process (Ma et al., 2020). Previous studies indicated that the reverse complementary sequences, like Alu sequences, may facilitate back-splicing by closing up the flanking introns together. Besides, m6A-enriched sites were easier to generate back-splicing as reported (Tang et al., 2020). Therefore, further experiments are needed to investigate the biogenesis of circRNAs. Here, we found that NOLC1 is also upregulated in PCa tissues using TCGA database, which is validated by a previous research (Kwon et al., 2020). We then illustrated that NF-kappaB could positively regulate linear NOLC1 mRNA and circNOLC1 expression in PCa. Previous study also exhibited that NOLC1 could be positively regulated by NF-kappaB and CREB (Gao et al., 2011). Through these results, we demonstrated that NF-kappaB could also regulate the expression of circRNAs, which gives a new insight in circRNAs biogenesis.

## Conclusion

**FIGURE 7:**
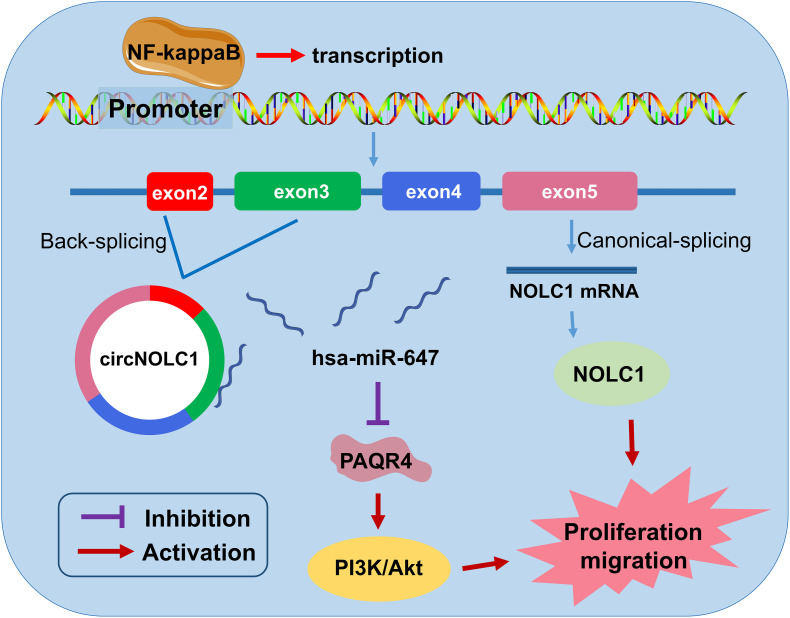
The biological function and mechanism of circNOLC1 in PCa. Schematic diagram exhibiting the biological function and mechanism of circNOLC1 in PCa.

In summary, we discovered a novel circRNA (circNOLC1) remarkably upregulated in PCa cells and tissues. Our results indicated that circNOLC1 participate in the malignant progression in PCa, mainly by sponging miR-647 to upregulate PAQR4 expression, thus activating the PI3K/Akt pathway. Also, NOLC1 and circNOLC1 can be regulated by NF-kappaB via binding to NOLC1 promoter sites. Our studies suggested that circNOLC1/PAQR4 axis could serve as a novel biomarker and therapeutic target for PCa (Figure 7).

## Data Availability Statement

The raw data supporting the conclusions of this article will be made available by the authors, without undue reservation.

## Ethics Statement

The studies involving human participants were reviewed and approved by the ethics committee of Zhujiang Hospital, Southern Medical University. The patients/participants provided their written informed consent to participate in this study. The animal study was reviewed and approved by the ethics committee of Zhujiang Hospital, Southern Medical University. Written informed consent was obtained from the individual(s) for the publication of any potentially identifiable images or data included in this article.

## Author Contributions

XM, DL, and CL conceived and designed the study. WC and SC carried out the experiments. TY, KW, and LZ collected and analyzed the clinical data. JL and XZ analyzed and interpreted the data. WC and DL wrote the manuscript. XM and DL executed the funding acquisition. All authors have reviewed and approved the final version of the manuscript.

## Conflict of Interest

The authors declare that the research was conducted in the absence of any commercial or financial relationships that could be construed as a potential conflict of interest.

## Footnotes

1 http://www.circbank.cn/

2 https://circinteractome.nia.nih.gov/

3 starbase.sysu.edu.cn/

4 http://consite.genereg.net/

## Supplementary Material

Supplementary Figure 1 Screening of circRNAs in PCa cells. **(A)** Heatmap of all differentially expressed circRNAs between PC3, DU145, C4-2, 22RV1, LNCaP, and RWPE1 cells. Red and green strips represent high and low expression, respectively. **(B–D)** Expression of hsa_circ_0018998, hsa_circ_0000735, hsa_circ_0050649, and hsa_circ_0002722 in five PCa cells and normal prostate cells.

Supplementary Figure 2 Knockdown of NOLC1 inhibits PCa cells proliferation and migration. **(A,B)** NOLC1 mRNA expression after knockdown circNOLC1 in DU145, C4-2, PC3. **(C)** RT-qPCR was performed to confirm the silencing efficiency of three siRNAs targeting NOLC1 in DU145 cells. **(D)** CCK8 was applied to exam the proliferation of DU145 cells after silencing the NOLC1. **(E)** Transwell migration assay was conducted to assess the cell migration ability in NOLC1-silenced DU145 cells.

## Abbreviations

circRNAs: circular RNAs
PCa: prostate cancer
qRT-PCR: quantitative real-time polymerase chain reaction
ceRNA: competing endogenous RNA
IHC: immunohistochemistry
NC: negative control
siRNA: small interfering RNA.
