# Supplementary figures and images for: Circular RNA CircNOLC1, Upregulated by NF-KappaB, Promotes the Progression of Prostate Cancer via miR-647/PAQR4 Axis
Source: Front Cell Dev Biol. 2021 Jan 8;8:624764. doi: 10.3389/fcell.2020.624764 (PMC7820754; doi:10.3389/fcell.2020.624764)

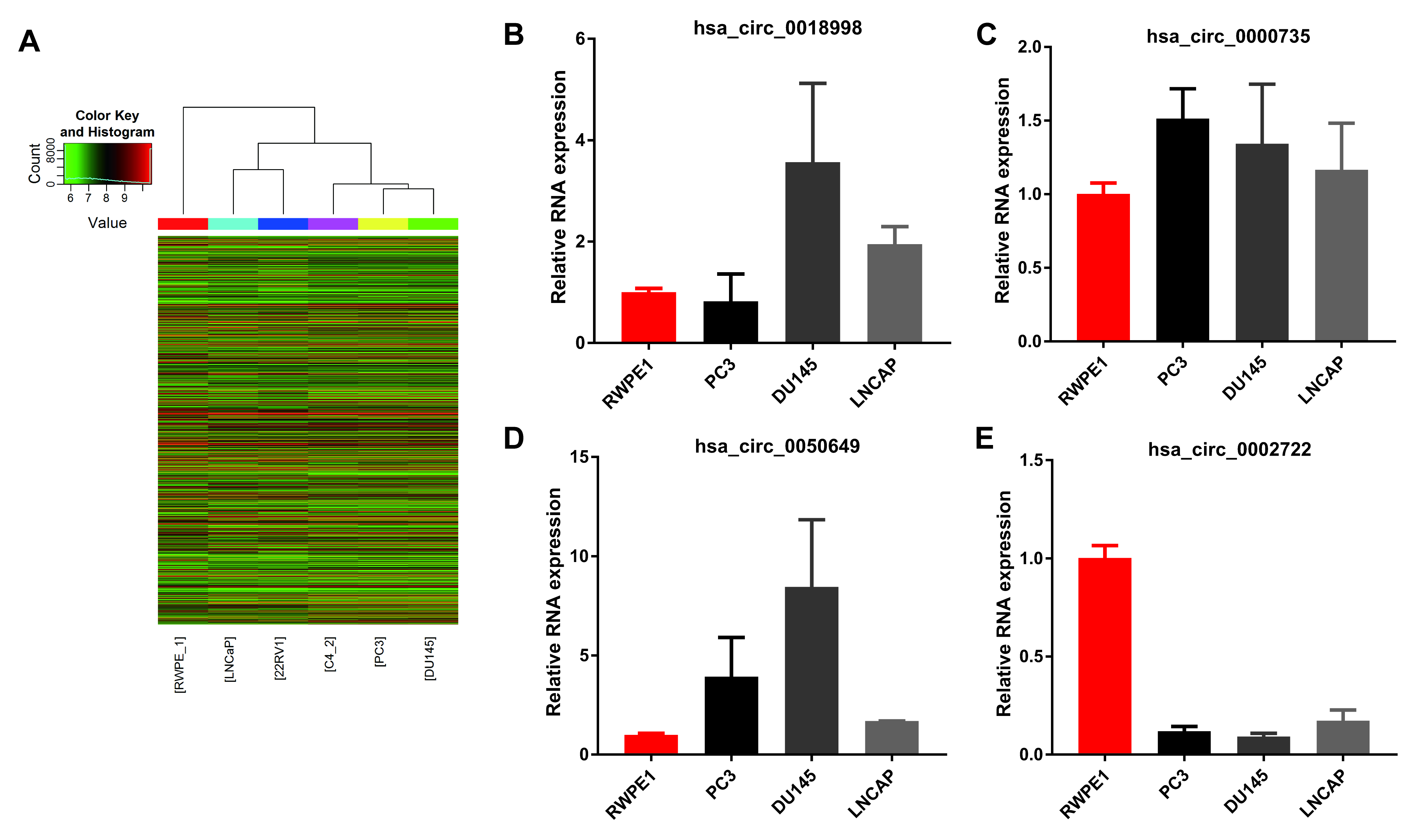

Supplement: Supplementary Figure 1 — Screening of circRNAs in PCa cells. (A) Heatmap of all differentially expressed circRNAs between PC3, DU145, C4-2, 22RV1, LNCaP, and RWPE1 cells. Red and green strips represent high and low expression, respectively. (B–D) Expression of hsa_circ_0018998, hsa_circ_0000735, hsa_circ_0050649, and hsa_circ_0002722 in five PCa cells and normal prostate cells. [file Image_1.TIF]

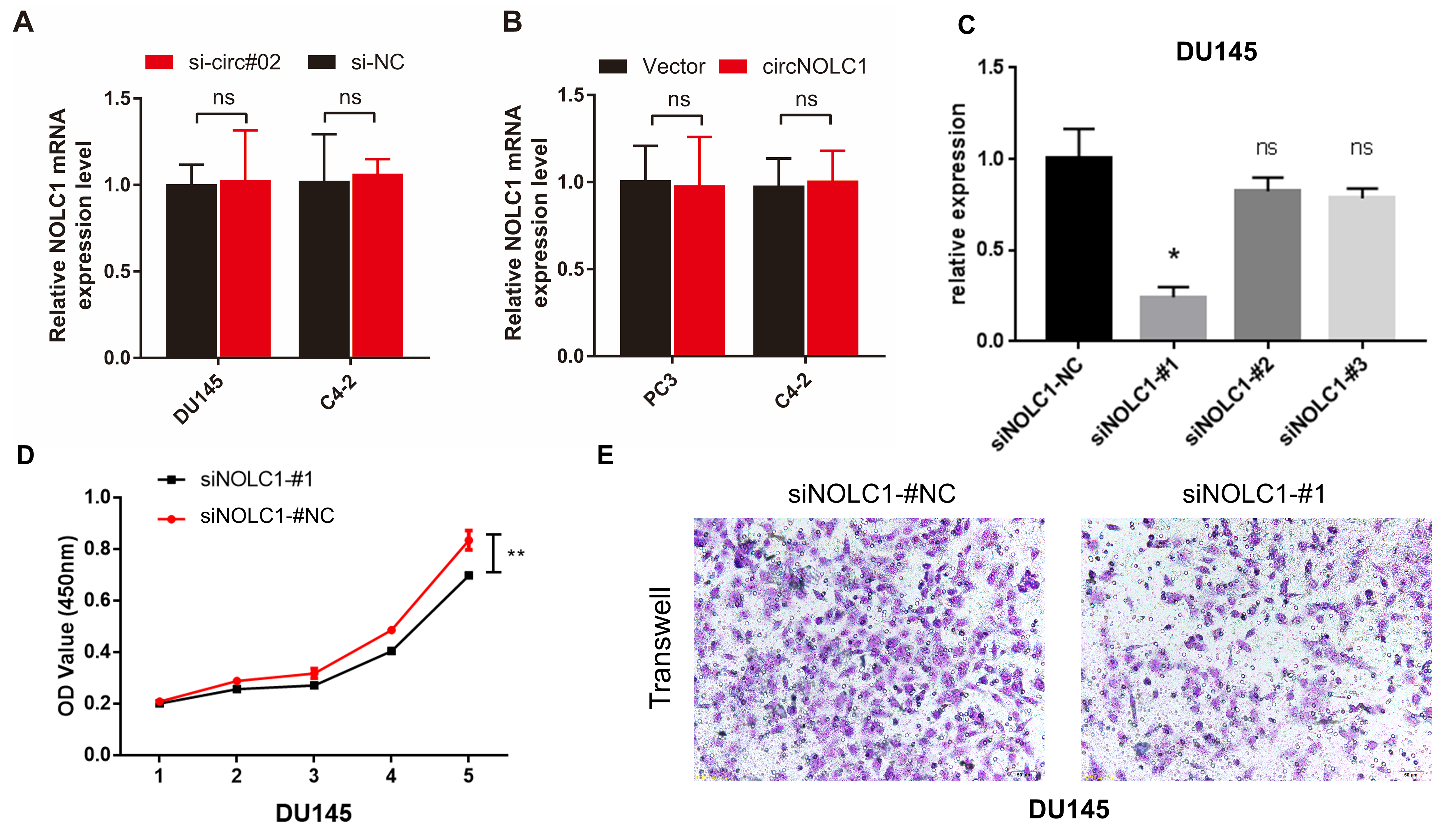

Supplement: Supplementary Figure 2 — Knockdown of NOLC1 inhibits PCa cells proliferation and migration. (A,B) NOLC1 mRNA expression after knockdown circNOLC1 in DU145, C4-2, PC3. (C) RT-qPCR was performed to confirm the silencing efficiency of three siRNAs targeting NOLC1 in DU145 cells. (D) CCK8 was applied to exam the proliferation of DU145 cells after silencing the NOLC1. (E) Transwell migration assay was conducted to assess the cell migration ability in NOLC1-silenced DU145 cells. [file Image_2.TIF]
